# The downregulation of lncRNA *EMX2OS* might independently predict shorter recurrence-free survival of classical papillary thyroid cancer

**DOI:** 10.1371/journal.pone.0209338

**Published:** 2018-12-21

**Authors:** Yi Gu, Chao Feng, Tong Liu, Bowei Zhang, Lan Yang

**Affiliations:** 1 Department of Vascular and Thyroid Surgery, Sichuan Academy of Medical Sciences and Sichuan Provincial People's Hospital, University of Electronic Science and Technology of China, Chengdu, Sichuan, China; 2 Department of Anatomy, Histology and Embryology, Chengdu University of Traditional Chinese Medicine, Chengdu, Sichuan, China; University of South Alabama Mitchell Cancer Institute, UNITED STATES

## Abstract

Homeobox protein Emx2 is a transcription factor that is encoded by the *EMX2* gene. In this study, using data from the Cancer Genome Atlas-Thyroid Cancer (TCGA-THCA), we aimed to examine the expression profile of *EMX2* and its antisense transcript *EMX2OS* in papillary thyroid cancer (PTC), their prognostic value and potential regulatory networks. Results showed that in the three variants of PTC, *EMX2* was significantly downregulated in classical PTC, while *EMX2OS* were significantly downregulated in follicular and classical PTC, compared with adjacent normal tissues. Kaplan-Meier survival curves showed that *EMX2* and *EMX2OS* expression was not related to RFS in follicular PTC. In comparison, the high *EMX2* or *EMX2OS* group were associated with better RFS compared with their respective low expression group in classical PTC (*p* = 0.007 and 0.004 respectively). Correlation analysis showed that *EMX2* and *EMX2OS* were highly co-expressed in PTC tissues (Spearman’s r = 0.83). By performing stepwise regression, we found that *EMX2OS* was better than *EMX2* in predicting RFS in classical PTC. Multivariate analysis confirmed that high *EMX2OS* expression was an independent indicator of favorable RFS in classical PTC (HR: 0.239, 95%CI: 0.100 = 0.569, *p* = 0.001), after adjustment of pathological stages and residual tumors. By performing in silico analysis, we found that the genes co-expressed with *EMX2* or *EMX2OS* were highly overlapped. KEGG pathway analysis showed that these genes were enriched in the ECM-receptor interaction, focal adhesion, and PI3K-Akt signaling, protein digestion and absorption and proteoglycans in cancer pathways, which are closely related to cancer initiation and progression. Based on the findings, we infer that decreased *EMX2OS* expression might be a valuable prognostic biomarker of unfavorable RFS in classical PTC.

## Introduction

Papillary Thyroid Cancer (PTC) is the dominant form of thyroid cancer and is usually indolent in progression [1, 2]. Although the patients with PTC usually have favorable OS (10-year OS > 95%) after standard treatment, a small proportion (5–20%) of patients have an increased risk of disease recurrence and distant metastasis, which result in aggressive diseases and lethal outcomes [3]. However, PTC is a not homogeneous disease, but has several different histological variants, including classical, follicular, and tall-cell, which have varying pathological and clinical implications [4]. Therefore, the prognostic value of a biomarker might vary in different histological variants.

Homeobox protein Emx2 is a transcription factor that is encoded by the *EMX2* gene. It is a critical gene modulating the formation of the central nervous system and urogenital system during embryonic development [5–7]. In the past decade, there are emerging evidence showed that *EMX2* also acts as a tumor suppressor gene in multiple cancers, such as lung cancer [8, 9], gastric cancer [10] and glioma [11]. Mechanistically, EMX2 acts as a negative regulator of the Wnt signaling pathway [10, 12]. Besides, it also suppresses the Epithelial-to-Mesenchymal Transition (EMT) of some cancer cells [8]. Preserved *EMX2* expression might also have potential prognostic value in terms of overall survival (OS) and recurrence-free survival (RFS) in lung adenocarcinoma [13] and lung squamous cell carcinoma [8]. One previous study found that *EMX2* and its antisense transcript *EMX2OS*, which is a long non-coding RNA (lncRNA), display coordinate expression in normal endometrium and endometrial tumor tissues [14]. Another following study confirmed that there might be a reciprocal *EMX2/EMX2OS* regulatory loop that is required for sustained transcription of *EMX2* [15], suggesting that *EMX2OS* might regulate the expression of *EMX2*.

In this study, using data from the Cancer Genome Atlas-Thyroid Cancer (TCGA-THCA), we examined the expression profile of *EMX2* and *EMX2OS* in PTC, their prognostic value and potential regulatory networks.

## Materials and methods

### Retrospective analysis using data from TCGA

The 3^rd^ level data of TCGA-THCA was acquired and downloaded by using the UCSC Xena browser (https://xenabrowser.net/). In TCGA-THCA, the biospecimens were examined by experienced pathologists to ensure the accuracy [16]. Follicular variant includes those tumors with 99% follicular patterned, while the tall cell variant refers to the cases with 50% or greater tall cell features [16]. Only the cases with primary tumor and without histological neoadjuvant therapy were included. The following clinicopathological data were downloaded for re-analysis, including age at initial pathologic diagnosis, gender, sample type, histological types, pathological stage, the presence of residual tumors, the history of radiation therapy, recurrence-free survival (RFS) status and RFS time (days) were obtained.

### Bioinformatic analysis using cBioPortal for cancer genomics and string

The genes that were at least moderately co-expressed with *EMX2OS* in PTC (Spearman’s r≥0.4) were identified using cBioPortal for Cancer Genomics [17]. Then, the potential molecular interaction network among the genes and the Kyoto Encyclopedia of Genes and Genomes (KEGG) pathways in which they are involved were identified using String 10.5 (https://string-db.org/), by setting 0.4 as the minimum required interaction score.

### Statistical analysis

Statistical analysis was performed by using GraphPad Prism 7.04 (GraphPad Inc., La Jolla, CA, USA) and SPSS 25.0 software package (SPSS Inc., Chicago, IL, USA). Welch’s unequal variance t-test was performed to examine the difference in *EMX2/EMX2OS* expression. The association between *EMX2OS* expression and the clinicopathological parameters in patients with classical PTC was assessed by using the Chi-squared test by two-sided Fisher’s exact test.

Kaplan-Meier curves of RFS were generated by setting the Youden Index of *EMX2/EMX2OS* expression in receiver operating characteristic curve (ROC) for recurrence detection as the cutoff. Log-rank test was conducted to examine the significance of the difference between the curves. Nonparametric Spearman’s correlation analysis was performed to assess the correlation between *EMX2* and *EMX2OS* expression. Stepwise regression was conducted to assess the value of *EMX2* and *EMX2OS* expression as predictive variables for recurrence in classical PTC. Univariate and multivariate Cox regression models were used to evaluate the prognostic significance of *EMX2OS* expression. *p*<0.05 was considered statistically significant. Data used for Cox regression analysis was provided in S1 Table.

## Results

### Both *EMX2* and *EMX2OS* expression were significantly downregulated in classical PTC compared with their adjacent normal tissues

**Fig 1:**
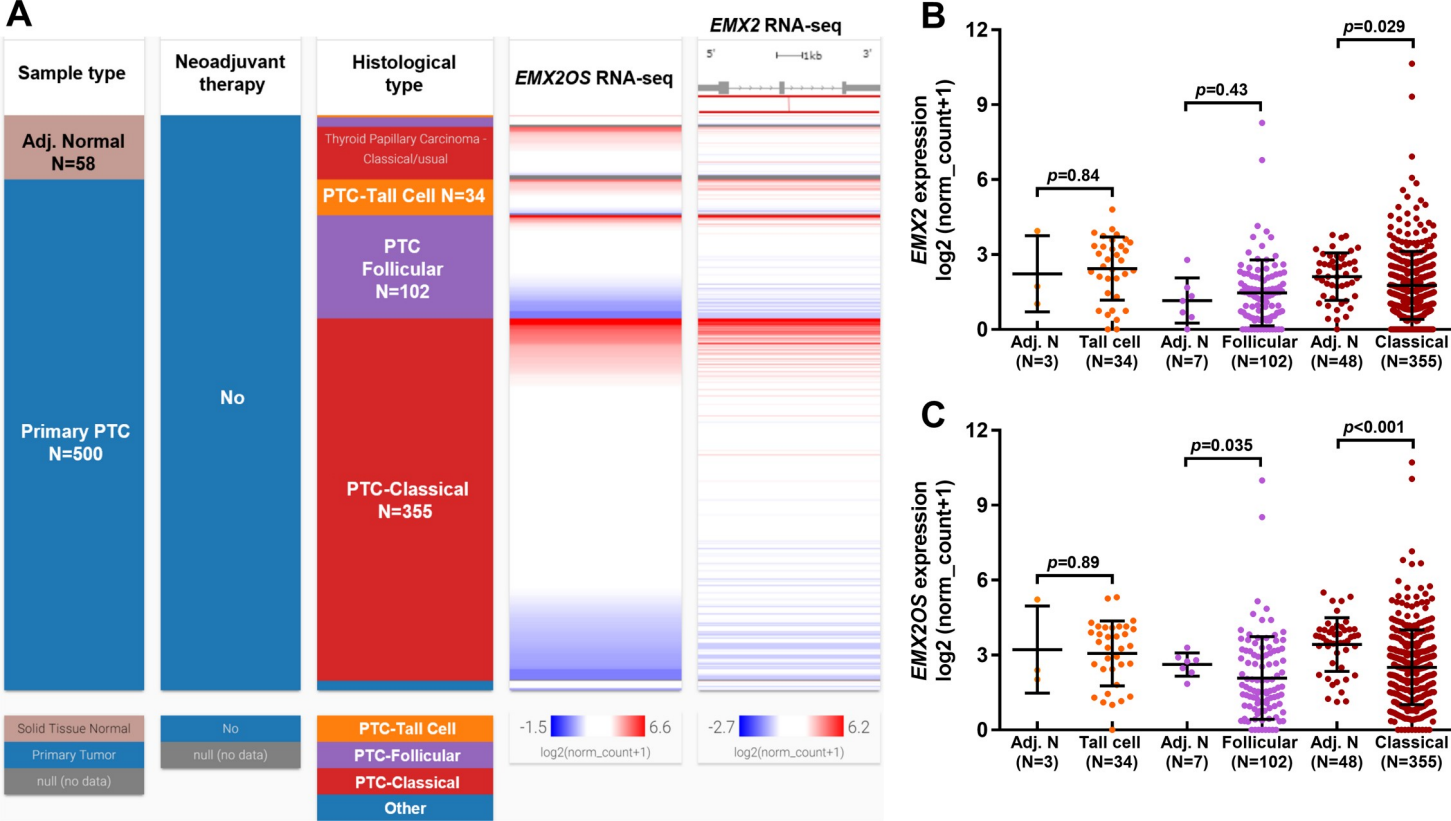
The expression profile of *EMX2* and *EMX2OS* in each variant of PTC compared and their adjacent normal tissues. **A-C.** Heatmap (A) and plots chart (B-C) showing the expression profile of *EMX2* (B) and *EMX2OS* (C) in each variant of PTC compared and their adjacent normal tissues. The number of tumor tissues and adjacent normal tissues were as indicated.

Based on the selection criteria, 500 cases of primary PTC and 58 cases of matched normal tissues were included. In the 500 primary tumor cases, there were 355 classical cases, 102 follicular cases, 34 tall cell cases and 9 cases not specified. Using the RNA-seq data of gene expression, we examined the expression of *EMX2* and *EMX2OS* in each variant of PTC (Fig 1A). Heatmap and the following plot charts showed that compared with adjacent normal tissues, classical PTC had significantly elevated *EMX2* expression (*p* = 0.029) (Fig 1B), while both follicular and classical PTC had significantly downregulated *EMX2OS* expression (*p* = 0.035 and *p*<0.001) (Fig 1C).

### Decreased *EMX2* and *EMX2OS* expression was associated with unfavorable RFS in classical PTC

**Fig 2:**
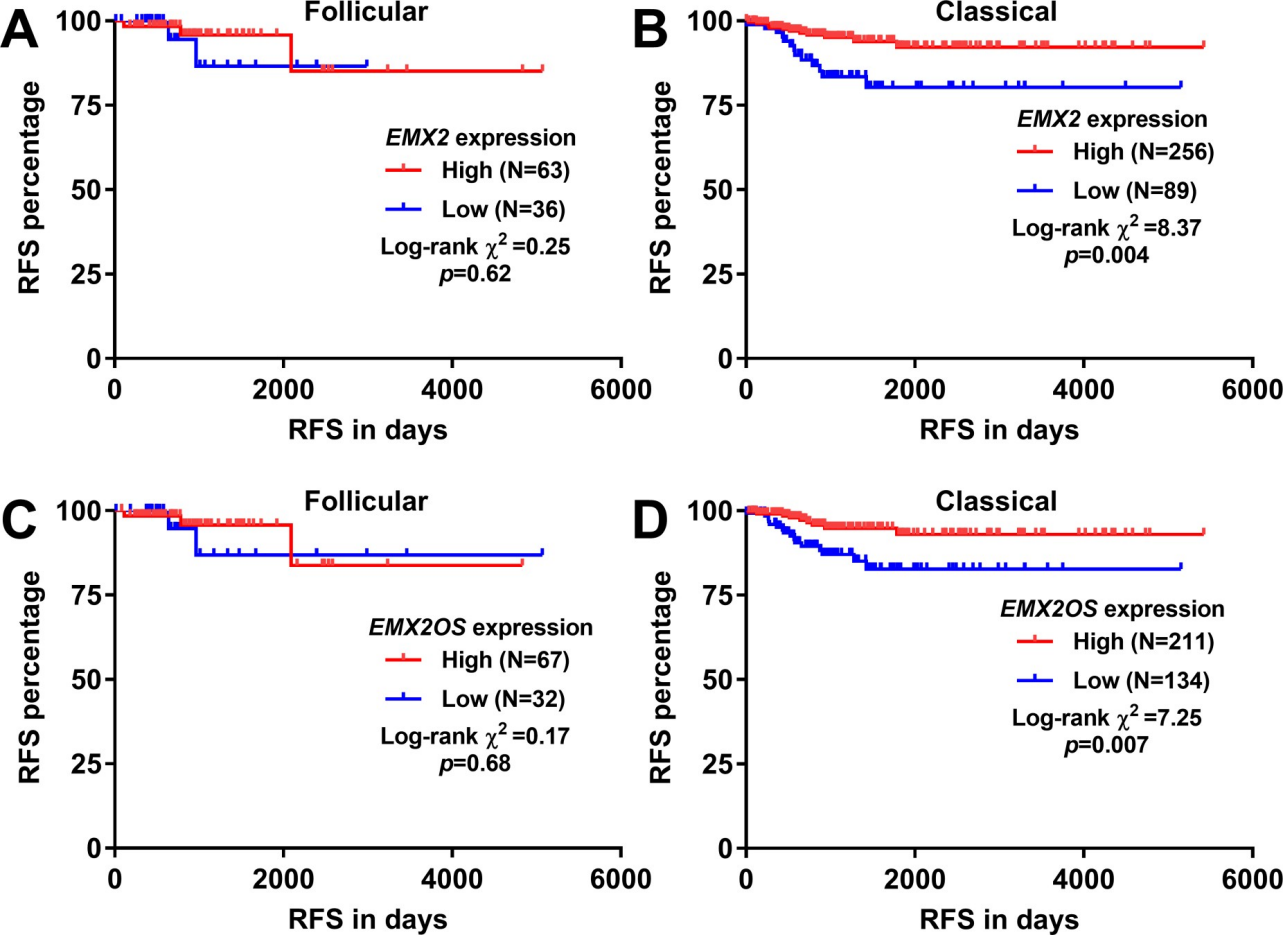
Decreased *EMX2* and *EMX2OS* expression was associated with unfavorable RFS in classical PTC, but not in follicular PTC. **A-D.** Kaplan-Meier curves of RFS in follicular (A and C) and classical (B and D) PTC. Patients were grouped according to the best cutoff of *EMX2* (A-B) and *EMX2OS* (C-D) expression in the ROC analysis for recurrence detection.

Then, we checked the association between the expression of *EMX2* and *EMX2OS* and RFS in follicular and classical PTC respectively. By using the best cutoff of *EMX2* and *EMX2OS* expression in ROC analysis for recurrence detection, we found that *EMX2* and *EMX2OS* expression was not related to RFS in the follicular variant of PTC (Fig 2A and 2C). In comparison, the classical PTC patients with high *EMX2* or *EMX2OS* expression had significantly better RFS compared with their respective low expression group (*p* = 0.004 and 0.007 respectively, Fig 2B and 2D).

**Fig 3:**
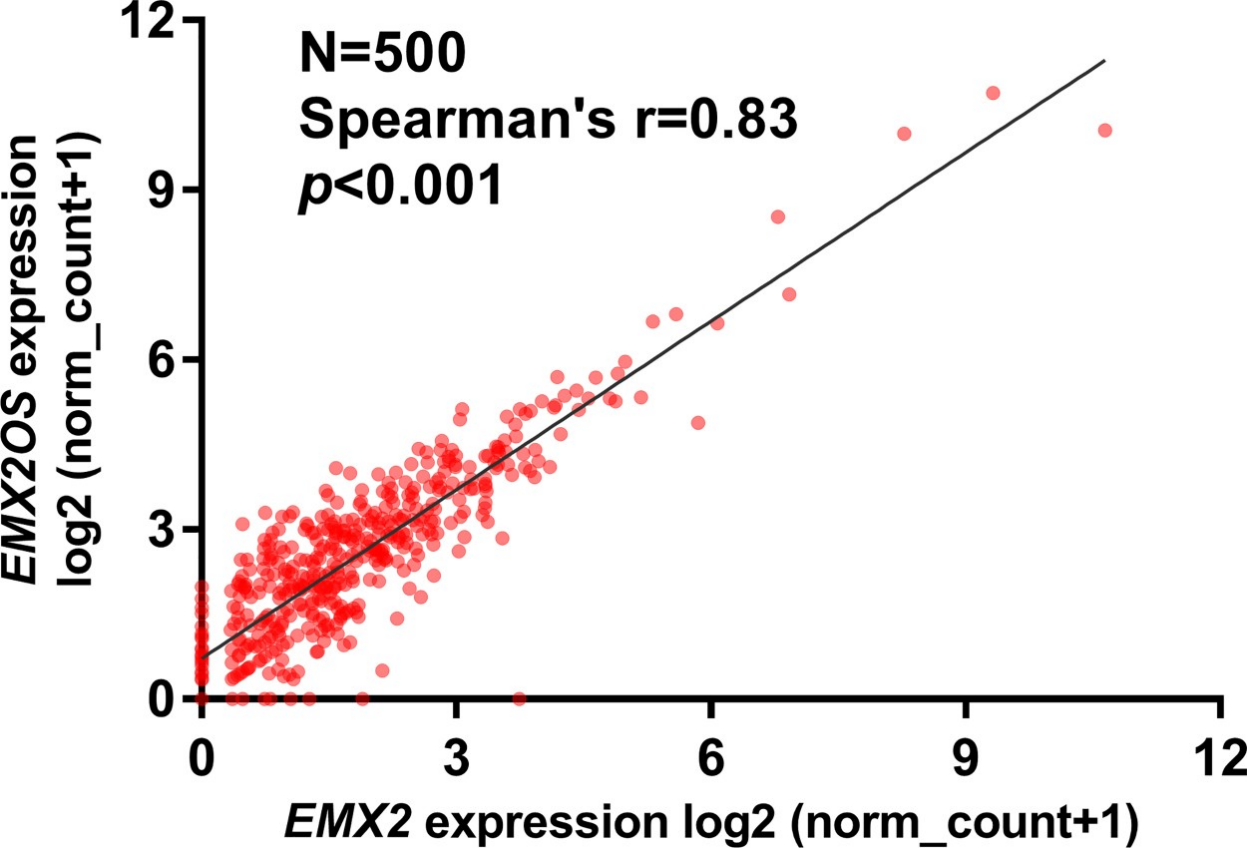
Correlation analysis between the expression of *EMX2* and *EMX2OS*. Spearman’s correlation between the expression of *EMX2* and *EMX2OS* in PTC cases (N = 500).

Then, we tried to explore their prognostic value in terms of RFS in classical PTC. Univariate analysis showed that advanced pathological stages, the presence of residual tumors, low *EMX2* expression and low *EMX2OS* are risk factors of shorter RFS (Table 1). Since *EMX2OS* is the opposite strand of *EMX2*, we further analyzed their expression correlation. Results showed that these two genes were highly co-expressed in PTC cases (N = 500, Spearman’s = 0.83) (Fig 3). Therefore, these two variables should not be included simultaneously in COX regression model. By performing stepwise regression, we found that *EMX2OS* is better than *EMX2* in predicting RFS in classical PTC (S1 Fig). Therefore, we only included *EMX2OS* in multivariate analysis. The clinicopathological parameters between the patients with high and low *EMX2OS* expression were summarized and compared in Table 2. Results showed that the high *EMX2OS* expression group had older age (47.89±1.13 *vs*. 43.83±1.34, *p* = 0.02) and a higher proportion of patients with pathological III/IV stage disease (36% vs. 25%, *p* = 0.044) and the presence of residual tumors (16% vs. 7%, *p* = 0.011), compared to the low *EMX2OS* expression group (Table 2). In multivariate analysis, we confirmed that high *EMX2OS* expression was an independent indicator of favorable RFS in classical PTC (HR: 0.239, 95%CI: 0.100 = 0.569, *p* = 0.001), after adjustment of pathological stages and residual tumors (Table 3).

**Table 1:** Univariate analysis of RFS in classical PTC patients.

Parameters	Univariate analysis			
	*p*	HR	95%CI (lower/upper)	
**Age** (Continuous)	0.263	1.013	0.990	1.037
**Gender**				
Male (N = 94)		1.000		
Female (N = 251)	0.487	0.742	0.320	1.721
**Clinical stage**				
I/II (N = 235)		1.000		
III/IV (N = 110)	**0.033**	2.348	1.069	5.155
**Radiotherapy**				
Yes (N = 205)		1.000		
No (N = 129)	0.333	0.650	0.271	1.557
**Residual tumors**				
No (N = 261)		1.000		
Yes (N = 43)	**0.015**	2.986	1.238	7.205
***EMX2* expression**				
Low (N = 89)		1.000		
High (N = 256)	**0.006**	0.332	0.152	0.728
***EMX2OS* expression**				
Low (N = 134)		1.000		
High (N = 211)	**0.010**	0.343	0.151	0.776

**Table 2:** The association between *EMX2OS* expression and the clinicopathological parameters in classical PTC patients.

		*EMX2OS* expression		*p* value
		High (N = 211)	Low (N = 134)	
**Age (Mean ± SD)**		47.89±1.13	43.83±1.34	**0.02**
**Gender**	Female	151(72%)	100(75%)	0.62
	Male	60(28%)	34(25%)	
**Pathological Stage**	III/IV	76(36%)	34(25%)	**0.044**
	I/II	135(64%)	100(75%)	
**Residual tumors**	R0	151(72%)	110(82%)	**0.011**
	R1/R2	34(16%)	9(7%)	
	RX/no data	26(12%)	15(11%)	
**Radiation therapy**	No	76(36%)	53(40%)	0.65
	Yes	126(60%)	79(59%)	
	No data	9(4%)	2(1%)	
**Recurrence status**	No	202(96%)	118(88%)	**0.01**
	Yes	9(4%)	16(12%)	

**Table 3:** Multivariate analysis of RFS in classical PTC patients.

Parameters	Multivariate analysis			
	*p*	HR	95%CI (lower/upper)	
**Clinical stage**				
I/II (N = 235)		1.000		
III/IV (N = 110)	**0.030**	2.524	1.091	5.837
**Residual tumors**				
No (N = 261)		1.000		
Yes (N = 43)	**0.018**	3.133	1.219	8.054
***EMX2OS* expression**				
Low (N = 134)		1.000		
High (N = 211)	**0.001**	0.239	0.100	0.569

### Bioinformatic analysis of the potential regulatory network of *EMX2* and *EMX2OS*

**Fig 4:**
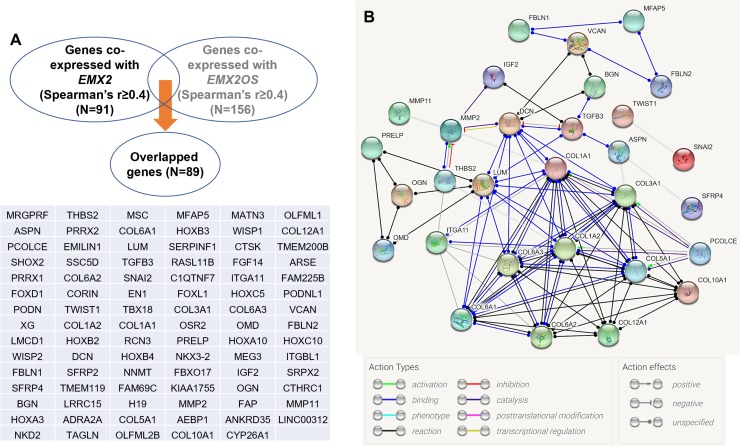
Bioinformatic analysis of the regulatory network of *EMX2* and *EMX2OS* co-expressed genes in PTC. **A.** The overlapping gene set between *EMX2* co-expressed with and *EMX2OS* co-expressed genes in PTC. Spearman’s r≥0.4 was set as the threshold. **B.** The potential regulatory network among the overlap genes. Analysis was performed using String 10.5.

**Fig 5:**
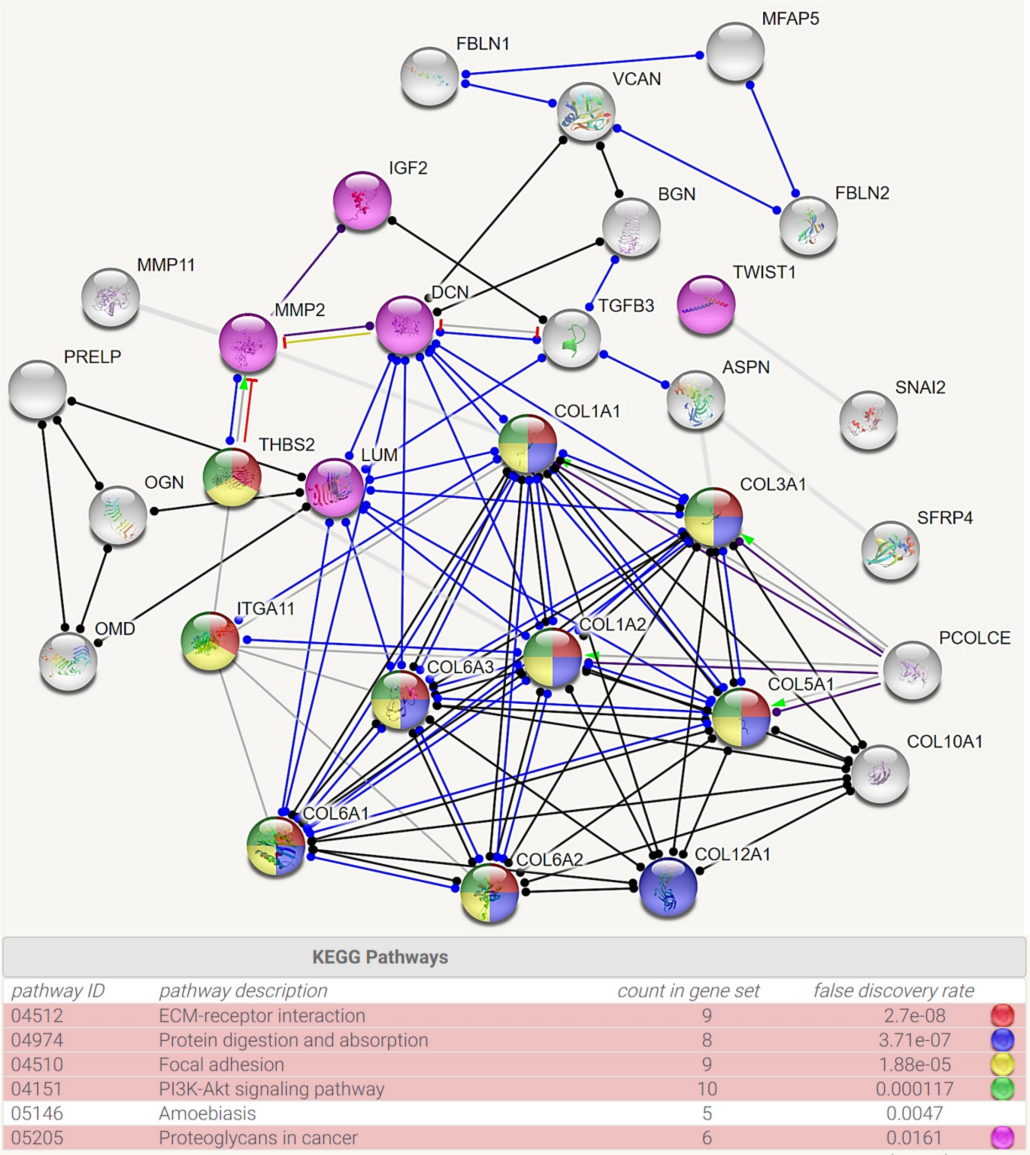
KEGG pathways of *EMX2* and *EMX2OS* co-expressed genes in PTC. The enrichment of the *EMX2* and *EMX2OS* co-expressed genes in PTC in KEGG pathways. Cancer-related pathways were colored.

To explore the potential regulatory network of *EMX2* and *EMX2OS*, we identified the genes that were at least moderately co-expressed with these two genes (Fig 4A). By setting Spearman’s r≥0.4 as the threshold, we found that there were 91 genes co-expressed with *EMX2* and 156 genes co-expressed with *EMX2OS* in PTC (Fig 4A). The overlapping set included 89 genes (Fig 4A). These findings suggest that *EMX2* and *EMX2OS* might have similar regulatory networks. Then, using STRING (version 10.5), we analyzed the potential regulatory network of the proteins encoded by these genes. Results showed that collagens (COL1A1, COL3A1, COL1A2, COL5A1, COL6A1, COL6A2, COL6A3, COL10A1, COL12A1 and PCOLCE), extracellular matrix proteins (LUM, DCN and MMP2) and cytokine TGFB3 are the key nodes in the network (Fig 4B). Then, we explored the KEGG pathway in which the genes might be enriched. Results showed that *THBS2*, *ITGA11*, *COL1A1*, *COL3A1*, *COL5A1*, *COL6A1*, *COL6A2* and *COL6A3* are involved in the ECM-receptor interaction, focal adhesion, and PI3K-Akt signaling pathway (Fig 5). *COL1A1*, *COL3A1*, *COL5A1*, *COL6A1*, *COL6A2*, *COL6A3*, and *COL12A1* are involved in the protein digestion and absorption pathway (Fig 5). *MMP2*, *IGF2*, *DCN*, *LUM* and *TWIST* are involved in proteoglycans in cancer pathway (Fig 5).

## Discussion

The tumor suppressor role of EMX2 has been characterized in several types of cancer. In lung cancer, *EMX2* is dramatically downregulated due to its promoter methylation, which enhances cell proliferation, invasive phenotypes and canonical WNT signaling [9]. Decreased *EMX2* mRNA expression is also a valuable prognostic marker of shorter OS and RFS in patients with lung adenocarcinoma [13]. Preserved *EMX2* expression is significantly associated with higher chemo-sensitivity and better OS in patients with lung squamous cell carcinoma [8]. In gastric cancer, adenovirus-mediated *EMX2* overexpression significantly inhibits cell proliferation and the Wnt signaling pathway both *in vitro* and in a gastric cancer xenograft model *in vivo* and prolongs the survival of tumor-bearing mice [10]. *EMX2* downregulation has been considered as a critical issue in the carcinogenesis of endometrial cancer and contributes to cancer progression [18, 19].

In this study, using RNA-seq study in TCGA-THCA, we found that both *EMX2* and its anti-sense transcript *EMX2OS* were downregulated in classical PTC compared with adjacent normal tissues. Kaplan-Meier survival curves showed that the patient group with low *EMX2* or *EMX2OS* expression had significantly worse RFS, compared to the group with high *EMX2* or *EMX2OS* expression. In univariate analysis, we confirmed that both high *EMX2* and *EMX2OS* expression were associated with better RFS (HR: 0.332, 95%CI: 0.152–0.728, *p* = 0.006; HR: 0.343, 95%CI: 0.151–0.776, *p* = 0.010 respectively). Since the coordinate expression between *EMX2* and *EMX2OS* has been reported in normal and cancerous human tissues [14], we then examined their correlation in PTC cases and confirmed that these two genes were very strongly co-expressed (Spearman’s r = 0.83). A series of previous studies demonstrated that the antisense transcript of a gene might not be transcriptional noise but may exert critical regulatory effect on the sense gene via diverse and complex mechanisms. For example, it may alter the epigenetic state of chromatin, via modulating the status of DNA methylation and/or histone modifications [20, 21]. Besides, it may also recruit transcription factors to certain regions of the sense genes, thereby modulating its transcription [22, 23]. In addition, the antisense transcript might also regulate the splicing and/or the half-life of its partner sense pre-mRNA [24, 25]. One previous study found that there might be a reciprocal *EMX2/EMX2OS* regulatory loop that is required for sustained transcription of *EMX2* in murine cortico-cerebral precursors [15], suggesting that *EMX2OS* might regulate the expression of *EMX2*. However, the exact mechanisms of the coordinately expressed *EMX2/EMX2OS* in PTC cells is worthy of future studies. The results of stepwise regression showed that *EMX2OS* is better than *EMX2* in predicting RFS in classical PTC. Multivariate analysis confirmed the independent prognostic value of *EMX2OS* expression in terms of RFS (HR: 0.239, 95%CI: 0.100 = 0.569, *p* = 0.001), after adjustment of pathological stages and residual tumors. These findings suggest that *EMX2OS* might serve as a valuable biomarker predicting RFS in classical PTC. One recent study found that preserved *SCN4B* might also be a prognostic marker of favorable RFS in classical PTC [26]. However, the authors had not explored the expression and prognostic value of *SCN4B* in other variants of PTC. In the current study, we found that although *EMX2OS* was downregulated in the follicular and classical variants, it only has prognostic value in classical variant. In addition, the prognostic of a mRNA might be discrepant with its protein expression [27–29]. The prognostic value of a lncRNA is not bothered by this phenomenon. Therefore, *EMX2OS* might be a specific biomarker in classical PTC.

Although the tumor suppressor role of *EMX2* might help to explain the association between *EMX2*/*EMX2OS* expression and better RFS in classical PTC, the exact regulatory effect of *EMX2*/*EMX2OS* in this variant have been not explored. To provide clues for future molecular studies, we tried to explore the potential regulatory network involving *EMX2* and *EMX2OS* in PTC by in silico analysis. Since *EMX2* is a transcription factor that might participate in a series of signaling pathways, we identified its co-expressed genes and compared with the co-expressed genes of *EMX2OS*. Their co-expressed genes were highly overlapped, suggesting that they might have similar regulatory networks. By performing network analysis, we found that some collagens, extracellular matrix-related proteins and cytokine are the key nodes in the network. KEGG pathway analysis showed that these genes were involved the ECM-receptor interaction, focal adhesion, and PI3K-Akt signaling, protein digestion and absorption and proteoglycans in cancer pathway. These pathways are all closely related to cancer initiation and progression. Therefore, it is meaningful to further explore the regulatory effect of *EMX2/EMX2OS* on the expression of these genes.

## Conclusion

Decreased *EMX2OS* expression might be a valuable prognostic biomarker of unfavorable RFS in classical PTC.

## Supporting information

S1 Fig Stepwise regression analysis of the value of *EMX2* and *EMX2OS* expression as predictive variables for recurrence in classical PTC. (DOCX)

S1 Table Data used for Cox regression analysis. (XLSX)
