# Supplementary figures and images for: The downregulation of lncRNA EMX2OS might independently predict shorter recurrence-free survival of classical papillary thyroid cancer
Source: PLoS One. 2018 Dec 21;13(12):e0209338. doi: 10.1371/journal.pone.0209338 (PMC6303026; doi:10.1371/journal.pone.0209338)

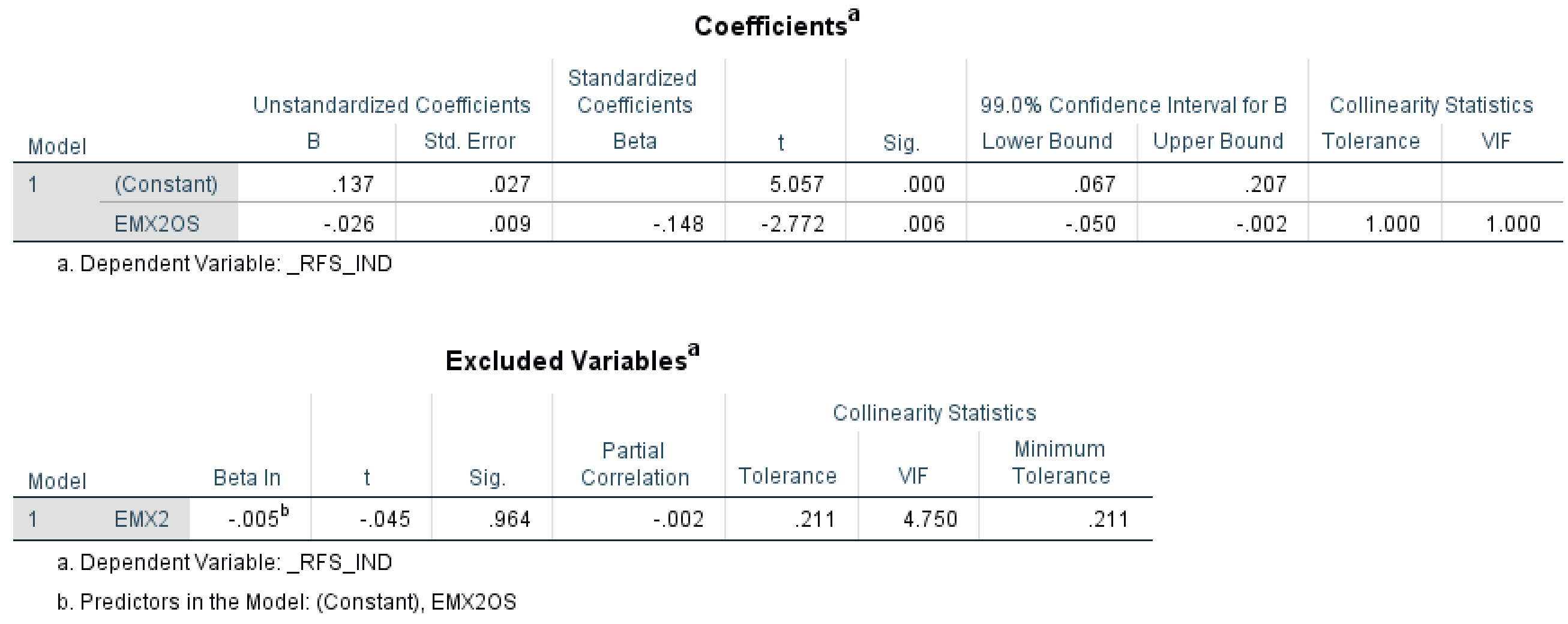

Supplement: S1 Fig — (DOCX) [file pone.0209338.s001.docx]
